# Erectile Dysfunction and Its Impact on Health-Related Quality of Life in Prostate Cancer Patients: A Multicenter Cross-Sectional Study from Pakistan

**DOI:** 10.3390/epidemiologia7010017

**Published:** 2026-01-27

**Authors:** Mateen Abbas, Márió Gajdács, Georgina Balogh, Sana Ahmed, Rabia Mahfooz, Abad Khan

**Affiliations:** 1 Department of Pharmacy Practice, Faculty of Pharmacy, Capital University of Science and Technology, Islamabad 44000, Pakistan; mateen.abbas@cust.edu.pk (M.A.); bph213117@cust.pk (R.M.); 2 Department of Public Health, Albert Szent-Györgyi Faculty of Medicine, University of Szeged, 6720 Szeged, Hungary; 3 Doctoral School of Experimental and Preventive Medicine, University of Szeged, 6720 Szeged, Hungary; balgeo@outlook.hu; 4 Department of Pharmacy, The University of Lahore, Lahore 54000, Pakistan; sana.ahmed@pharm.uol.edu.pk; 5 Department of Pharmacy, University of Swab, Swabi 23531, Pakistan; drabadkhan@uoswabi.edu.pk

**Keywords:** prostate cancer, erectile dysfunction, health-related quality of life, HRQoL, Pakistan

## Abstract

Background/Objectives: Prostate cancer (PC) is one of the most commonly diagnosed malignancies globally; depending on the treatment strategy used, erectile dysfunction (ED) is a frequently reported adverse outcome among PC patients. The current study evaluated ED prevalence among Pakistani PC patients and its effects on physical, psychological, and social well-being, aiming to address critical gaps in survivorship care for this population. Methods: A cross-sectional, multicenter, observational, questionnaire-based study was conducted in Rawalpindi and Islamabad, Pakistan, from February to April 2025. Health-related quality of life (HRQoL) among PC patients was measured using the Short Form Health Survey 36 (SF-36), while ED prevalence and severity were assessed by the International Index of Erectile Function (IIEF) instrument. Results: Among *N* = 400 PC patients, surgical treatments predominated (radical prostatectomy: 61.0%; *n* = 244), while hormonal (androgen-deprivation therapy: 31.5%; *n* = 126) and chemotherapy (23.3%; *n* = 93) were also commonly utilized. ED experience was high among PC patients in the erectile function (40.8%; *n* = 163) and in the intercourse satisfaction (45.0%; *n* = 180) domains; these showed moderately strong and significant positive correlations across all SF-36 domains, particularly physical functioning (r = 0.52; *p* < 0.001) and social functioning (r = 0.49; *p* < 0.001). Regression analysis confirmed sexual function domains explained 60% of HRQoL variance (adjusted R^2^ = 0.60). Conclusions: This study reveals high rates of treatment-related ED—and its biopsychosocial impact–among Pakistani PC patients, with significant negative impacts on HRQoL. The findings underscore the urgent need to integrate sexual health management into standard oncological care practices to improve holistic patient outcomes.

## 1. Introduction

Malignant disorders are the second major cause of overall mortality across the globe, while in people under 65 years of age, cancers are responsible for the highest number of years of lost life (YLLs) [1]. According to the most recent (2023) estimations of the Global Burden of Disease (GBD) Study, 18.5 million new cases and 10.4 million cancer-related deaths were recorded globally [2]. Prostate cancer (PC) is one of the most commonly diagnosed malignancies among men worldwide, accounting for over 1.4 million new cases and an estimated 375,000 deaths annually, as per the 2020 data of the Global Cancer Observatory (GCO) [3]. PC constitutes ~15% of all male cancer cases and is the most common incident cancer in over 100 countries, particularly in developed regions, such as North America, Europe, Australia, and New Zealand [4]. This has also been underscored by GBD estimates, showing an 8.8% increase in age-standardized incidence rates of PC in the period between 1990 and 2023, respectively [2]. In a Pakistani context, PC is among the ten most frequently diagnosed cancers and the third most widespread genitourinary malignancy among the male population [5,6]. In contrast, South Asian countries have historically reported lower incidence rates of PC; however, emerging data suggest a rising burden of disease in this region, including Pakistan [7]. This increase has been attributed to various factors and exposures, including longer life expectancy, growing levels of urbanization, lifestyle changes (both in diet and physical activity), and improved diagnostic capabilities [8]. According to the National Cancer Registry Data from the Pakistan Atomic Energy Cancer Registry (PAECR)—which is responsible for data collection and analysis on a national level in 18 cancer hospitals—show a growing trend of PC, particularly among males ages 60 years and above [9]. Despite this rise in PC incidence, the lack of a comprehensive national cancer registry, limited public awareness pertaining to the disease, and cultural stigma continue to hinder accurate reporting and timely diagnosis in Pakistan [10].

Erectile dysfunction (ED), characterized by the consistent inability to achieve or maintain an erection sufficient for satisfactory sexual performance, is a frequently reported adverse outcome among PC patients [6,11]. Prostate cancer-associated ED is largely driven by vascular and endothelial dysfunction, as well as fibrosis of the corpus cavernosum. Emerging therapeutic strategies often aim to restore penile vascularization, modulate fibrotic processes, and improve smooth muscle–endothelial signaling. Autologous immune cell-based regenerative therapies have shown potential in improving vasculogenic erectile dysfunction by promoting endothelial repair and tissue regeneration [12]. Additionally, pharmacological agents, such as vericiguat, a soluble guanylate cyclase stimulator, have demonstrated vascular benefits in cardiovascular disease and may have translational potential for ED management in prostate cancer survivors [13]. These strategies represent a growing focus on integrating vascular-targeted and regenerative approaches to improve post-treatment sexual function. Depending on the treatment modality utilized for their care, the prevalence of ED in PC survivors ranges from 30% to 85% [9]. Treatments, such as radical prostatectomy, radiation therapy, and androgen deprivation therapy (ADT), often disrupt neurovascular pathways or suppress hormonal function, leading to varying degrees of erectile impairment [7]. ED, however, is not solely a physiological concern; it may considerably affect patients’ emotional well-being, interpersonal relationships, confidence, and overall health-related quality of life (HRQoL) [8]. HRQoL refers to the multidimensional aspects of an individuals’ health and well-being, encompassing physical, psychological, and social functioning over time [14]. Several studies have highlighted that sexual dysfunction is one of the most distressing post-treatment challenges among PC survivors, often associated (or co-occurring) with depression, social withdrawal, and reduced self-worth [9]. In Pakistan, where discussions surrounding sexual health are often stigmatized, the psychological and relational impacts of ED may be further amplified [15]. The cultural reluctance to address sexual dysfunction openly, both within families and in the context of clinical consultations, results in the underreporting and clinical undertreatment of ED [10]. Additionally, there is a dearth of epidemiological evidence from a local perspective on the extent of this issue, and few studies have examined how ED affects HRQoL among PC patients in Pakistani healthcare settings [11]. With this in mind, the aim of the study was to assess the prevalence of ED in PC patients in Pakistan and evaluate its impact on HRQoL among these patients. Our study aims to provide additional information to the scientific literature related to a culturally sensitive health topic among males to allow for its more effective management within the Pakistani healthcare system.

## 2. Materials and Methods

### 2.1. Objectives

The primary objective of this study was to determine the prevalence and severity of ED, assessed using the erectile function (EF) domain of the International Index of Erectile Function (IIEF) instrument, among PC patients. Secondary objectives included evaluating HRQoL using the Short Form Health Survey 36 (SF-36) and examining the association between ED severity and relevant HRQoL domains.

### 2.2. Study Design, Setting, and Duration

A cross-sectional, multicenter, observational, questionnaire-based study was performed in Pakistan. A non-probability–convenience–sampling technique was employed to select respondents willing to participate in our study during the time of the data collection. The study was carried out between February and April 2025.

### 2.3. Study Population, Inclusion and Exclusion Criteria

Data collection was performed across three tertiary care hospitals and oncology treatment centers in the “twin cities” of Pakistan—located in the Punjab province—where ED patients may receive their initial diagnoses, treatment, and follow-up care [16]. Tertiary care hospitals situated in the region are large, specialized healthcare institutions that provide advanced medical and surgical treatment, which had a bed capacity ranging from 400 to over 1000 beds, allowing them to manage a large number of patients, including critical and referred cases from smaller facilities across northern Punjab, Khyber Pakhtunkhwa, and Azad Kashmir. Patients were approached during their routine visits to the facilities as mentioned above to participate in our research. The inclusion criteria of the study population were the following: (i) community-dwelling males aged 18 years or older, (ii) diagnosis with histologically-confirmed prostate cancer, irrespective of disease stage or (expected) treatment modality, (iii) patients who had completed primary treatment (e.g., surgery, radiotherapy, hormonal therapy, chemotherapy) or were on active surveillance/watchful waiting, with treatment completion or initiation occurring at least three months prior to enrollment, (iv) having a minimum of primary education, (v) individuals who had the mental competency to consent to participation in the study, (vi) able to read and comprehend English, allowing for the filling-out of the data collection instruments, (vii) willing to take part in the study voluntarily, and provided written informed consent. The exclusion criteria of the study were as follows: (i) people who did not wish to take part in the study (due to refusal of consent, or due to language, literacy, or physical limitations), (ii) those who were unable to comprehend English to a sufficient degree to fill out the questionnaire, (iii) individuals with a history of psychiatric illnesses or cognitive impairments, affecting their ability to understand or complete the questionnaires, (iv) patients with a known history of ED before the diagnosis of PC, (v) patients with neurological disorders independently associated with erectile dysfunction (e.g., Parkinson’s disease, spinal cord injury, multiple sclerosis), (vi) those receiving medications known to impair sexual function (e.g., selective serotonin reuptake inhibitors, antipsychotics), (vii) patients who had not completed their treatment at least three months prior to the study or were undergoing ongoing acute-phase treatment.

Sample size determination was performed using the Raosoft Sample Size Calculator (http://www.raosoft.com/samplesize.html; accessed on: 1 March 2024), based on the below formula (1):

(1) $$n=N\frac{x}{\left(N-1\right)E2+x}$$

where “*x*” is the expected response rate, “*E*” is the acceptable margin of error (5%, i.e., the required level of confidence was 95%), the population (N) was set at 20,000 (default setting of the software), and the expected response rate was set at 50% [17]. As a result, the required sample size—to ensure adequate statistical power to detect meaningful associations between studied variables—of *n* = 377 was set for the completion of this study. During the study period, 462 PC patients were approached to take part in the study, out of which 430 were eligible; in total, *N* = 400 consented to participate and were enrolled in the final sample. Reasons for non-participation included refusal (*n* = 18), language or literacy barriers (*n* = 9), and incomplete questionnaires (*n* = 3), respectively.

### 2.4. Data Collection Tool, Validated Instruments Used

A paper-based, interviewer-administered, 55-item standardized questionnaire was used for data collection, which included the following subdomains and validated instruments: (i) socio-demographic characteristics, including the PC patients’ age, type, and highest level of educational attainment, occupational status, source of medical financial support, (ii) characteristics of PC treatment among our respondents (i.e., receipt of surgical, hormonal, chemotherapy, immunotherapy, and cryotherapy), (iii) HRQoL was measured using the SF-36, a validated and standardized instrument, which comprises 36-items, assessing QoL in eight core domains, irrespective of the specific medical condition affecting the respondent, i.e., physical functioning (PF; 10-items), role limitations due to physical health (RP; 4-items), bodily pain (BP; 2-items), general health perceptions (GH; 5-items), vitality (VT; 4-items), social functioning (SF; 2-items), role limitations due to emotional problems (RE; 3-items) and mental health (MH; 5-items). The SF-36 provides scores between 0 and 100 across the eight QoL domains (the higher the better), using standard scoring algorithms, offering a comprehensive view of overall well-being [18]. (iv) ED was assessed using the IIEF, a validated and standardized instrument, which comprises 15-items measured on a 5-point Likert scale, assessing male sexual function—or the presence and severity of ED in five core domains, i.e., erectile function (EF; 6-items), orgasmic function (OF; 2-items), sexual desire (SD; 2-items), intercourse satisfaction (IS; 3-items) and overall satisfaction (OS; 2-items). The IIEF-15 yields a total score ranging from 15 to 75, with higher scores indicating better sexual function [19]. Interpretation of the EF domain—used to determine the prevalence and severity of ED–was carried out based on the following cut-off values: score 26–30: no ED, score 22–25: mild ED, score 17–21: mild-to-moderate ED, score 11–16: moderate ED, score 10 or below: severe ED.

### 2.5. Statistical Analysis

After data collection, questionnaire data was transferred to Statistical Package for Social Sciences v.26.0 (SPSS; IBM Corp., Endicott, NY, USA) for analysis. During descriptive statistics, all continuous variables were expressed as means (with standard deviations [SD] and ranges), while categorical variables were described as frequencies and percentages (*n*, %). Normality testing of data was carried out using quantile-quantile (Q-Q) diagrams and Kolmogorov–Smirnov tests. Associations among ED scores and HRQoL components were examined using Pearson correlation coefficients (r_p_). The strength of the relationship between variables was described as follows: |r_p_| < 0.3 was denoted as a weak correlation, 0.3 < |r_p_| < 0.5 as a moderate correlation, and 0.5 < |r_p_| < 0.85 as a strong correlation [20,21]. Given the multiple correlation analyses performed between SF-36 domains (8 domains) and IIEF domains (5 domains), a total of 40 (5 × 8) correlation tests were conducted. To control for the increased risk of type I error accumulation due to multiple testing, a Bonferroni correction was applied. Accordingly, the adjusted significance threshold was set at *p* < 0.00125 (0.05 (α)/40). Correlations meeting this adjusted threshold were considered statistically robust, while associations significant at the *p* < 0.05 threshold were interpreted with caution as exploratory findings. Additionally, to assess the independent association between ED and HRQoL, a multiple linear regression analysis was performed. The dependent variable was the overall HRQoL score derived from the SF-36 instrument. Independent variables included the five IIEF domains, i.e., EF, OF, SD, IS, and OS. The model was adjusted for potential confounders, including age, educational level, employment status, type of PC treatment received (surgery, hormonal therapy, chemotherapy), and time since treatment completion. Regression coefficients (β), standard errors (SE), and *p*-values were reported. During regression analyses, statistical significance was set at *p* < 0.05.

### 2.6. Ethical Considerations

The study was conducted in accordance with the Declaration of Helsinki (1975, last revised in 2024) [22] and national and institutional ethical standards. Ethical approval for this study was obtained from the Ethical Committee of the Faculty of Pharmacy, Capital University of Science and Technology, Islamabad, Pakistan (approval ID: CUST-PH-20-24; date of approval: 5 January 2024). Furthermore, approvals were also obtained from the heads of the healthcare facilities in Pakistan, where data collection was conducted during the study period.

Before taking part, all participants were provided with both verbal and written information regarding the study’s purpose, procedures, potential risks, and benefits. Both verbal and written informed consent was obtained from each participant, ensuring that they understood their right to participate voluntarily and their right to withdraw at any time without any negative consequences. The confidentiality and anonymity of participants were strictly maintained throughout the study. Participant identifiers were replaced with unique codes, and all data were stored securely in compliance with data protection regulations. Special care was taken to ensure that sensitive issues related to sexual health and ED were handled with respect and professionalism, in alignment with the cultural context of the study population. The participants did not receive any incentives (monetary or otherwise) to take part in the study. Considering potential cultural sensitivities around sexual health, additional time was given to answer questions and ensure understanding, reinforcing the voluntary nature of participation. However, participants were informed that they could seek counseling or medical advice if they experienced any discomfort or distress related to the HRQoL or ED questions of the survey.

### 2.7. Reporting Guidelines

This manuscript adheres to the Strengthening the Reporting of Observational Studies in Epidemiology (STROBE) guidelines for cross-sectional studies [23]; the STROBE checklist is provided in Supplementary Material S1.

## 3. Results

### 3.1. Socio-Demographic Characteristics

Overall, *N* = 400 PC patients from the relevant healthcare institutions agreed to take part in our study. A detailed description of the socio-demographic characteristics of the participants is shown in Table 1. The study sample consisted of participants across various age groups, with the highest proportion of PC patients in the 60–69-year-old (25.75%; *n* = 103) and 50–59-year-old (22.50%; *n* = 90) age ranges, respectively. However, participants aged 18–39 years represented a smaller proportion of the cohort (19.25%; *n* = 77) and had histologically confirmed PC diagnoses. Most cases in this age group were evaluated for suspected hereditary or early-onset disease and were managed at tertiary oncology centers. The majority of participants had a bachelor’s degree (37.00%; *n* = 150), while 21.75% (*n* = 87) had a postgraduate education. In terms of employment status, government employees were the largest group (29.25%; *n* = 117), followed by private sector employees (23.25%; *n* = 93); 13.00% (*n* = 52) of PC patients were retirees. Regarding medical financial support, half of the participants (50.25%, *n* = 201) were self-financed, while 22.00% (*n* = 88) received governmental support (Table 1).

### 3.2. Treatment Characteristics for PC Patients Among the Study Population

The treatment options among the study participants were diverse (summarized in Table 2), with surgical treatments being one of the most commonly utilized (61.00%; *n* = 244); out of surgical strategies, radical prostatectomy was the most frequently used option, accounting for 38.25% (*n* = 153) of PC patients in our sample. Hormonal therapy was another key treatment approach (64.75%; *n* = 259), with androgen deprivation therapy (ADT) being the most commonly prescribed for PC patients (31.50%; *n* = 126) of participants. Luteinizing hormone-releasing hormone (LHRH) agonists were prescribed to 17.75% (*n* = 71), while anti-androgens were used by 11.75% (*n* = 47) of our sample. Cytotoxic chemotherapy was administered to a smaller proportion of PC patients (23.25%; *n* = 93), with docetaxel being the most common chemotherapeutic drug used (15.25%; *n* = 61). Immunotherapy (3.25%; *n* = 13) and cryoablation of the prostate (3.75%; *n* = 15) were utilized in the context of a very small portion of participants (Table 2).

### 3.3. Health-Related Quality of Life (HRQoL) and Sexual Function Among PC Patients

HRQoL scores–measured via the SF-36 instrument–across various domains revealed a range of physical and mental health functioning in our sample of PC patients; the mean scores for each of the domains were summarized in Table 3. Overall, low domain-specific HRQoL was observed for 12.00% (*n* = 48), 18.00% (*n* = 72), 22.00% (*n* = 88), 25.00% (*n* = 100), 50.00% (*n* = 200), 15.00% (*n* = 60), 40.00% (*n* = 160), and 35.00% (*n* = 140) for the PF, RP, BP, GH, VT, SF, RE, and MH subdomains, respectively.

The ED scores across the relevant domains showed varying levels of impairment in PC patients. The mean score for ED was 12.5 ± 5.43, with 40.75% of patients reporting the experience of ED in this domain. OF had a mean score of 4.35 ± 1.60, with 34.75% of patients experiencing dysfunction. SD scored a mean of 4.65 ± 1.50, with 30.0% of patients reporting issues in this area. IS had the lowest mean score at 5.57 ± 2.79, with 45.0% of patients experiencing difficulties. OS had a mean score of 4.00 ± 2.05, with 42.0% of patients reporting unsatisfactory sexual experiences (Table 4). Based on the scores of the EF domain, 2.75% (*n* = 11) experienced mild, 6.25% (*n* = 25) had mild-to-moderate, 17.00% (*n* = 68) showed moderate, and 14.75% (*n* = 59) showed severe ED, respectively.

### 3.4. Inferential Statistics Between HRQoL and ED Among PC Patients

As a part of our study, correlational analyses were carried out between individual SF-36 domains and subdomains of the IIEF instrument—corresponding to various aspects of ED—in our PC sample (Table 5). PF was strongly and positively correlated with EF (r = 0.52, *p* = 0.001), OF (r = 0.49, *p* = 0.002), SD (r = 0.47, *p* = 0.003), IS (r = 0.55, *p* < 0.001), and OS (r = 0.58, *p* < 0.001), respectively. RP also demonstrated strong and significant correlations with EF (r = 0.48, *p* = 0.002), OF (r = 0.50, *p* = 0.001), SD (r = 0.45, *p* = 0.005), IS (r = 0.52, *p* = 0.001), and OS (r = 0.56, *p* < 0.001), respectively. BP demonstrated moderate-to-strong and significant correlations with EF (r = 0.39, *p* = 0.04), OF (r = 0.42, *p* = 0.01), IS (r = 0.44, *p* = 0.003), and OS (r = 0.50, *p* < 0.001), respectively. GH showed moderate-to-strong and significant correlations with EF (r = 0.44, *p* = 0.003), OF (r = 0.46, *p* = 0.002), SD (r = 0.40, *p* = 0.04), IS (r = 0.47, *p* = 0.002), and OS (r = 0.51, *p* = 0.001), respectively. VT showed moderate-to-strong and significant correlations with EF (r = 0.33, *p* = 0.03), OF (r = 0.35, *p* = 0.02), SD (r = 0.30, *p* = 0.045), IS (r = 0.40, *p* = 0.005), and OS (r = 0.45, *p* < 0.001), respectively. SF showed moderate-to-strong and significant correlations with EF (r = 0.49, *p* = 0.001), OF (r = 0.47, *p* = 0.002), SD (r = 0.43, *p* = 0.004), IS (r = 0.51, *p* < 0.001), and OS (r = 0.54, *p* < 0.001), respectively. BP demonstrated moderately strong, significant correlations with EF (r = 0.38, *p* = 0.048), OF (r = 0.42, *p* = 0.01), IS (r = 0.41, *p* = 0.005), and OS (r = 0.46, *p* < 0.001), respectively. SF showed moderate-to-strong and significant correlations with EF (r = 0.41, *p* = 0.004), OF (r = 0.43, *p* = 0.002), SD (r = 0.39, *p* = 0.03), IS (r = 0.45, *p* = 0.001), and OS (r = 0.50, *p* < 0.001), respectively (Table 5).

After applying Bonferroni correction for multiple correlations, the most notable correlations between HRQoL domains and IIEF subdomains, particularly those involving PF, RE, SF, and OS, remained significant below the adjusted significance threshold, indicating robust associations between ED severity and HRQoL.

Table 6 presents the results of the multiple linear regression model, where the SF-36 HRQoL scores were set as outcome variables, while the IIEF domains were set as covariates. Our unadjusted model (R^2^ = 0.62, F-statistic = 32.85, constant: 42.30) indicated significant relationships between the various IIEF domains of sexual function and the HRQoL scores, as follows: EF β: 0.45 (*p* = 0.01), OF β: 0.38 (*p* = 0.001), SD β: 0.22 (*p* = 0.046), IS β: 0.50 (*p* = 0.01), and OS β: 0.55 (*p* = 0.01), respectively; The adjusted model statistics showed an R^2^ of 0.60, indicating that ~60% of the variance in the HRQoL scores may be explained by the domains of sexual function: after adjustment for age, treatment category, time since treatment, and diabetes status, EF (β = 0.41, *p* = 0.01), OF (β = 0.32, *p* = 0.004), IS (β = 0.44, *p* = 0.01), and OS (β = 0.49, *p* = 0.01) remained significantly associated with HRQoL.

## 4. Discussion

PC has become one of the most common cancers globally among males, corresponding to a substantial burden of disease [3]; while incident cases have shown a steady increase in recent years, age-standardized mortality rates between 1990 and 2023 have decreased by 17.2% [5]. This notable decrease in mortality may be associated with developments in diagnostic technologies (e.g., advanced imaging, such as multiparametric MRIs, targeted biopsies, and the use of more reliable biomarkers), surgical and radiation techniques, and personalized pharmacotherapy [24]. Nonetheless, it must be noted that the availability of these current medical technologies is often limited to high-income regions, leading to disparities in PC-associated care and health outcomes across the globe [25].

The present study employed a multicentric cross-sectional design to explore the prevalence of ED and its impact on HRQoL among PC patients in Pakistan. Our findings confirmed that ED is not only highly prevalent in this population but also notably affects various dimensions of HRQoL, particularly those related to emotional, social, and mental well-being. From a biopsychosocial perspective, ED is more than a physiological consequence of PC or its treatment; it is a psychosocial condition with far-reaching consequences [26]. The finding that PC patients with moderate-to-severe ED scored significantly lower across emotional and social functioning domains supports this model [27]. This aligns with previous studies, which highlighted that the emotional consequences of ED often surpass its physical implications, with patients reporting heightened anxiety, diminished self-worth, and social withdrawal [28,29]. Our results also underscored that even those with mild ED reported HRQoL impairments, albeit to a lesser degree. This supports the framework proposed by the Cognitive Appraisal Theory, which posits that individuals evaluate stressors based on personal significance and coping resources [30]. In Pakistan’s very particular cultural context—where masculinity and sexual performance are closely tied to identity and self-concept—the perception of ED as a loss of manhood may exacerbate psychological distress, irrespective of ED severity [31].

A critical interpretation of our findings must consider the sociocultural construction of masculinity. In South Asian societies—including Pakistan—discussing sexual dysfunction still remains taboo, and masculinity is often equated with sexual virility [32]. This cultural silence may hinder patients from expressing distress or seeking support, thereby worsening HRQoL outcomes. Moreover, the stigma attached to ED may lead to feelings of shame, isolation, and social withdrawal, which were reflected in our findings through significantly lower emotional well-being and social engagement scores among ED sufferers [33]. This aligns with Connell’s Theory of Hegemonic Masculinity, which suggests that men internalize dominant masculine ideals, making them less likely to seek help for conditions like ED that challenge their externally perceived masculinity. Thus, our results not only confirm the physical burden of ED but also highlight a significant gendered health disparity, which may often be masked by cultural norms [34].

From a clinical standpoint, our findings reinforce earlier work by Resnick et al. (2013), which showed that PC treatment, particularly prostatectomy and androgen deprivation therapy (ADT), which were highly prevalent in our sample, are strongly associated with long-term sexual dysfunction [35,36]. While these modalities may improve oncological outcomes, they frequently result in irreversible ED, an adverse event often under-communicated in pre-treatment counseling [37]. This gap contributes to unmet expectations, post-treatment dissatisfaction, and deteriorating HRQoL [38]. In line with Shared Decision-Making Theory, our findings support the need for transparent, patient-centered discussions about the sexual side effects of prostate cancer therapies [39]. Patients should be empowered to weigh trade-offs between survival and sexual function (and more broadly, HRQoL), especially given the increasing life expectancy of prostate cancer survivors [40]. While international guidelines advocate for integrating sexual rehabilitation into survivorship care (NCCN, 2023) [41], implementation in Pakistan remains limited [42]. From a Health Belief Model (HBM) standpoint, patients may not seek help for ED due to perceived stigma, low perceived benefits of treatment, or lack of cues to action [43]. Our findings highlight an urgent need for public health campaigns that normalize sexual health discussions, challenge stigmas, and empower men affected by ED associated with PC treatments to seek support without fear of judgment [44,45].

The inclusion of multiple centers during our study has enhanced the generalizability of findings across diverse patient populations and clinical environments. Nevertheless, it is also important to acknowledge the study’s limitations, including its cross-sectional design and convenience sampling (introducing selection bias), which precludes causal inferences between ED and HRQoL. Furthermore, self-reported data—collected through the means of questionnaires—may also be influenced by social desirability bias, especially in culturally sensitive domains like sexuality. Moreover, the requirement for minimum primary education and English literacy was applied to ensure valid self-completion and accurate interpretation of standardized instruments (SF-36 and IIEF), which have not been formally validated in Urdu within oncology populations in Pakistan. This restriction may have resulted in a study population with a study cohort that was skewed toward higher educational attainment, which may not fully reflect the broader PC population in Pakistan, particularly in under-resourced public hospitals and lower-literacy populations. Furthermore, we acknowledge that sexual recovery is undoubtedly influenced not only by the patient but also by partner dynamics. Spousal attitudes, expectations, and responses to ED may significantly impact sexual function and HRQoL, but these factors were not captured in this study. Future research should incorporate partner-reported outcomes to better understand the social and relational context of ED recovery. Future longitudinal studies should explore the trajectory of ED and HRQoL over time and assess the effectiveness of various psychosocial interventions in improving outcomes. Moreover, in-depth analysis via qualitative research could illuminate the nuanced experiences and coping mechanisms of PC patients, offering culturally informed insights into how ED is perceived, internalized, and managed in South Asian contexts.

## 5. Conclusions

The current study highlights the biopsychosocial impact of ED on PC patients in Pakistan, revealing not only its high prevalence but also its detrimental effects on HRQoL, particularly in emotional, social, and mental health domains. The strong correlations between HRQoL domains and sexual dysfunction measures highlight the interplay between physical and psychological well-being. At the same time, the sociocultural stigma surrounding ED exacerbates distress by reinforcing masculine ideals that discourage help-seeking behavior. Despite international guidelines advocating for sexual rehabilitation in survivorship care, Pakistan’s healthcare system lacks structured support, leaving patients to navigate unmet expectations and deteriorating HRQoL post-treatment. These findings demand urgent clinical and sociocultural interventions, including patient-centered counseling on treatment side effects, destigmatization of sexual dysfunction, and integration of psychosocial support into oncology care.

## Figures and Tables

**Table 1:** Summary of the demographic characteristics of the PC patients participating in our study (*N* = 400).

Variable	Frequency (*n*)	Percentage (%)
Age group (Years)		
18–29	37	9.25
30–39	40	10.00
40–49	70	17.50
50–59	90	22.50
60–69	103	25.75
70 and over	60	15.00
Highest level of educational attainment		
Below primary school/Primary school	52	13.00
Secondary school	61	15.25
Bachelors (BSc/BA)	150	37.00
Masters (MSc/MA) or postgraduate	87	21.75
Islamic education	50	12.50
Employment Status		
Government employee	117	29.25
Private sector employee	93	23.25
Self-employed	60	15.00
Unemployed	78	19.05
Retired	52	13.00
Medical financial support		
Self-financed	201	50.25
Government supported	88	22.00
Insurance	59	14.75
Charity	52	13.00

**Table 2:** Treatment modalities received by the PC patients during disease course (*N* = 400).

Type of Treatment (Main Groups)	Frequency (*n*) *	Percentage (%) *
Surgical treatment	244	61.00
Radical prostatectomy	153	38.25
Laparoscopic prostatectomy	57	14.25
Transurethral resection of the prostate (TURP)	34	8.50
Hormonal therapy	259	64.75
Androgen deprivation therapy (ADT)	126	31.50
Luteinizing hormone-releasing hormone (LHRH) agonists	71	17.75
Anti-androgens	47	11.75
Bilateral orchiectomy	15	3.75
Chemotherapy	93	23.25
Docetaxel	61	15.25
Cabazitaxel	19	4.75
Mitoxantrone	13	3.25
Immunotherapy	13	3.25
Sipuleucel-T	5	1.25
Immune checkpoint inhibitors (e.g., pembrolizumab, nivolumab)	8	2.00
Cryotherapy	15	3.75
Cryoablation of the Prostate	15	3.75

* Note: Patients may have received more than one type of treatment during their disease course; therefore, the total number of treatments and the cumulative percentages exceeded the total study population (*N* = 400) or 100.0%. Each treatment category reflects the number and proportion of patients who were given that specific therapy, regardless of overlap with other treatment modalities.

**Table 3:** Health-related quality of life (HRQoL) measured via SF-36 among PC patients participating in our study (*N* = 400).

Domain	Score (Mean ± SD)	Score Range (Min–Max)	Patients Reporting Low HRQoL in the Specific Domain (%, *n*)
Physical functioning (PF)	75.5 ± 12.3	40–100	12.00% (*n* = 48)
Role limitations due to physical health (RP)	72.4 ± 15.7	30–100	18.00% (*n* = 72)
Bodily pain (BP)	68.3 ± 14.1	35–95	22.00% (*n* = 88)
General health (GH)	70.2 ± 13.5	40–90	25.00% (*n* = 100)
Vitality (VT)	45.3 ± 16.2	10–70	50.00% (*n* = 200)
Social functioning (SF)	79.1 ± 10.5	50–100	15.00%; (*n* = 60)
Role limitations due to emotional problems (RE)	56.7 ± 20.3	20–95	40.00% (*n* = 160)
Mental health (MH)	58.9 ± 14.8	30–85	35.00% (*n* = 140)
Average Quality of Life Score	65.8 ± 13.4	-	-

HRQoL: health-related quality of life; SD: standard deviation.

**Table 4:** Prevalence of ED measured via the IIEF instrument among PC patients participating in our study (*N* = 400).

Domain	Score (Mean ± SD)	Score Range (Min–Max)	Patients Reporting ED Issues in the Specific Domain (%, *n*)
Erectile function (EF)	12.5 ± 5.43	7–27	40.75% (*n* = 163)
Orgasmic function (OF)	4.35 ± 1.60	3–6	34.75% (*n* = 139)
Sexual desire (SD)	4.65 ± 1.50	3–6	30.00% (*n* = 120)
Intercourse satisfaction (IS)	5.57 ± 2.79	3–8	45.00% (*n* = 180)
Overall satisfaction (OS)	4.00 ± 2.05	2–6	42.00% (*n* = 168)

**Table 5:** Correlation coefficient between SF-36 domain and IIEF domains was measured among PC patients participating in our study.

		IIEF Domains				
	Pearson-Correlation Coefficents (r_p_) (*p*-Values)	Erectile Function (EF)	Orgasmic Function (OF)	Sexual Desire (SD)	Intercourse Satisfaction (IS)	Overall Satisfaction (OS)
SF-36 domains	Physical Functioning (PF)	0.52 ** **(0.001)**	0.49 * (0.002)	0.47 * (0.003)	0.55 ** **(<0.001)**	0.58 ** **(<0.001)**
	Role limitations due to physical health (RP)	0.48 * (0.002)	0.50 ** **(0.001)**	0.45 * (0.005)	0.52 ** **(0.001)**	0.56 ** **(<0.001)**
	Bodily pain (BP)	0.39 * (0.04)	0.42 * (0.01)	0.36 (0.06)	0.44 * (0.003)	0.50 ** **(<0.001)**
	General health (GH)	0.44 * (0.003)	0.46 * (0.002)	0.40 * (0.04)	0.47 * (0.002)	0.51 ** **(0.001)**
	Vitality (VT)	0.33 * (0.03)	0.35 * (0.02)	0.30 * (0.045)	0.40 * (0.005)	0.45 ** **(<0.001)**
	Social functioning (SF)	0.49 ** **(0.001)**	0.47 * (0.002)	0.43 * (0.004)	0.51 ** **(<0.001)**	0.54 ** **(<0.001)**
	Role limitations due to emotional problems (RE)	0.38 * (0.048)	0.42 * (0.01)	0.34 (0.06)	0.41 * (0.005)	0.46 ** **(<0.001)**
	Mental health (MH)	0.41 * (0.004)	0.43 * (0.002)	0.39 * (0.03)	0.45 ** **(0.001)**	0.50 ** **(<0.001)**

Pearson-correlation coefficients (r_p_) are presented along with corresponding *p*-values in parentheses. Correlations significant at the 5% level (*p* < 0.05) are marked with a single asterisk (*), while those significant at the adjusted significance threshold (*p* < 0.00125) are marked with two asterisks (**) and in **boldface**.

**Table 6:** Multiple linear regression model describing the relationship between HRQoL based on SF-36 and sexual function based on IIEF domain measures among PC patients participating in our study.

	Model 1 *		Model 2 **	
Variables	Unadjusted β (SE)	*p*-Value	Adjusted β (SE)	*p*-Value
Erectile function (EF)	0.45 (0.09)	**0.01**	0.41 (0.10)	**0.01**
Orgasmic function (OF)	0.38 (0.10)	**0.001**	0.32 (0.11)	**0.004**
Sexual desire (SD)	0.22 (0.11)	**0.046**	0.18 (0.12)	0.08
Intercourse satisfaction (IS)	0.50 (0.08)	**0.01**	0.44 (0.09)	**0.01**
Overall Satisfaction (OS)	0.55 (0.07)	**0.01**	0.49 (0.08)	**0.01**
Constant	42.30 (3.20)	**0.01**	40.10 (3.50)	**0.01**

* Model 1: Unadjusted multiple linear regression including all IIEF domains; ** Model 2: Adjusted model for age, educational level, employment status, diabetes status, type of prostate cancer treatment, and time since treatment completion; β values are standardized regression coefficients; SE: standard error; Unadjusted R^2^ = 0.62; adjusted R^2^ = 0.60; F = 32.85; *p* < 0.001. *p*-values < 0.05 were denoted in **boldface**.

## Data Availability

The dataset is available from the Corresponding author on reasonable request.

## Supplementary Materials

The following supporting information can be downloaded at: https://www.mdpi.com/article/10.3390/epidemiologia7010017/s1, Supplementary Material S1: STROBE checklist.
